# RepeatsDB in 2021: improved data and extended classification for protein tandem repeat structures

**DOI:** 10.1093/nar/gkaa1097

**Published:** 2020-11-25

**Authors:** Lisanna Paladin, Martina Bevilacqua, Sara Errigo, Damiano Piovesan, Ivan Mičetić, Marco Necci, Alexander Miguel Monzon, Maria Laura Fabre, Jose Luis Lopez, Juliet F Nilsson, Javier Rios, Pablo Lorenzano Menna, Maia Cabrera, Martin Gonzalez Buitron, Mariane Gonçalves Kulik, Sebastian Fernandez-Alberti, Maria Silvina Fornasari, Gustavo Parisi, Antonio Lagares, Layla Hirsh, Miguel A Andrade-Navarro, Andrey V Kajava, Silvio C E Tosatto

**Affiliations:** Dept. of Biomedical Sciences, University of Padua, Via Ugo Bassi 58/B, Padua 35121, Italy; Dept. of Biomedical Sciences, University of Padua, Via Ugo Bassi 58/B, Padua 35121, Italy; Dept. of Biomedical Sciences, University of Padua, Via Ugo Bassi 58/B, Padua 35121, Italy; Dept. of Biomedical Sciences, University of Padua, Via Ugo Bassi 58/B, Padua 35121, Italy; Dept. of Biomedical Sciences, University of Padua, Via Ugo Bassi 58/B, Padua 35121, Italy; Dept. of Biomedical Sciences, University of Padua, Via Ugo Bassi 58/B, Padua 35121, Italy; Dept. of Biomedical Sciences, University of Padua, Via Ugo Bassi 58/B, Padua 35121, Italy; IBBM-CONICET, Dept. of Biological Sciences, La Plata National University, 49 y 115, 1900 La Plata, Argentina; IBBM-CONICET, Dept. of Biological Sciences, La Plata National University, 49 y 115, 1900 La Plata, Argentina; IBBM-CONICET, Dept. of Biological Sciences, La Plata National University, 49 y 115, 1900 La Plata, Argentina; Dept. of Science and Technology, National University of Quilmes, Roque Sáenz Peña 352, Bernal, Buenos Aires, Argentina; Dept. of Science and Technology, National University of Quilmes, Roque Sáenz Peña 352, Bernal, Buenos Aires, Argentina; Dept. of Science and Technology, National University of Quilmes, Roque Sáenz Peña 352, Bernal, Buenos Aires, Argentina; Dept. of Science and Technology, National University of Quilmes, Roque Sáenz Peña 352, Bernal, Buenos Aires, Argentina; Institute of Organismic and Molecular Evolution, Faculty of Biology, Johannes Gutenberg University of Mainz, Hans-Dieter-Hüsch-Weg 15, 55128 Mainz, Germany; Dept. of Science and Technology, National University of Quilmes, Roque Sáenz Peña 352, Bernal, Buenos Aires, Argentina; Dept. of Science and Technology, National University of Quilmes, Roque Sáenz Peña 352, Bernal, Buenos Aires, Argentina; Dept. of Science and Technology, National University of Quilmes, Roque Sáenz Peña 352, Bernal, Buenos Aires, Argentina; IBBM-CONICET, Dept. of Biological Sciences, La Plata National University, 49 y 115, 1900 La Plata, Argentina; Dept. of Engineering, Faculty of Science and Engineering, Pontifical Catholic University of Peru, Av. Universitaria 1801 San Miguel, Lima 32, Lima, Peru; Institute of Organismic and Molecular Evolution, Faculty of Biology, Johannes Gutenberg University of Mainz, Hans-Dieter-Hüsch-Weg 15, 55128 Mainz, Germany; Centre de Recherche en Biologie cellulaire de Montpellier, UMR 5237, CNRS, Univ. Montpellier, Montpellier, France; Dept. of Biomedical Sciences, University of Padua, Via Ugo Bassi 58/B, Padua 35121, Italy

## Abstract

The RepeatsDB database (URL: https://repeatsdb.org/) provides annotations and classification for protein tandem repeat structures from the Protein Data Bank (PDB). Protein tandem repeats are ubiquitous in all branches of the tree of life. The accumulation of solved repeat structures provides new possibilities for classification and detection, but also increasing the need for annotation. Here we present RepeatsDB 3.0, which addresses these challenges and presents an extended classification scheme. The major conceptual change compared to the previous version is the hierarchical classification combining top levels based solely on structural similarity (Class > Topology > Fold) with two new levels (Clan > Family) requiring sequence similarity and describing repeat motifs in collaboration with Pfam. Data growth has been addressed with improved mechanisms for browsing the classification hierarchy. A new UniProt-centric view unifies the increasingly frequent annotation of structures from identical or similar sequences. This update of RepeatsDB aligns with our commitment to develop a resource that extracts, organizes and distributes specialized information on tandem repeat protein structures.

## INTRODUCTION

The world of proteins is so diverse in their amino acid sequences, structural states and functions that in order to navigate efficiently between them we need their systematic classification and annotation. Although all known three-dimensional protein structures can be found in the Protein Data Bank (PDB) (1), significant efforts have been undertaken to further classify these structures. The best known protein structural classification databases, CATH (2) and SCOP (3), put the secondary structure of proteins at the forefront of their classification. This concept has led to a simple hierarchical classification of most protein structures, especially those with globular structures. Over the last two decades, a number of non-globular structures have been determined, containing tandem repeats (TRs) in their sequence and structure (4–6). These structures show unexpected structural similarities inconsistent with the usual classification schemas (7,8).

In search of a more harmonious classification for repeat proteins, RepeatsDB adopts a simple solution mainly based on repeat unit length (6). A repeat unit is the smallest structural building block forming the repeat region (9). The repeat region may include insertions, i.e. non-repeated segments occurring either inside a single repeat unit or between consecutive repeats. The protein repeat sequences can be described by two parameters: period and number of units/repeats. The period, or repeat length, is the number of amino acids contained in each repeat and this feature supported the design of the first TR classification schema. This classification allows better categorization of TR-containing proteins by common structural and functional characteristics and facilitates a better understanding of evolutionary mechanisms. TR-containing proteins are considerably diverse, ranging from the repetition of a single amino acid to repetitive domains of 100 or more residues. Depending on repeat length, protein structures are subdivided into five classes: (i) crystalline aggregates formed by regions with one or two residue long repeats; (ii) fibrous structures stabilized by inter-chain interactions with 3–7 residue repeats; (iii) elongated structures with repeats of 5–40 residues where repetitive units require one another to maintain structure; (iv) closed (not elongated) structures with repeats of 30–60 residues where repetitive units need one another and are arranged in a circular manner; (v) ‘beads on a string’ repeats with typically over 50 residues, which are large enough to fold independently into stable domains.

In order to automatically detect repetitive elements in protein structures, different types of approaches have been implemented. They include feature-based learning methods (RAPHAEL (10) and ConSole (11)), structural space tiling (12), Fourier analysis (13), wavelet transforms (14) and signal analysis methods (DAVROS (15), CE-Symm (16) and TAPO (17)). RepeatsDB expands manually curated unit annotations using the RepeatsDB-lite algorithm (18), a novel version of the previous ReUPred method (19). RepetasDB-lite is a template-based method which exploits manually curated knowledge available in RepeatsDB. Another tool, RAPHAEL (10), is used to calculate the repeat period. During the years, RepeatsDB has been expanded, revised and improved. Since version 2 (20), an improved classification schema and high quality annotations, i.e. unit definition, are available for all entries. RepeatsDB data has been used to analyse their structural arrangement (16) and folding pathways (21,22), to discuss repeats in genomes (23,24) and to benchmark new methods for repeat detection (16,18,25,26).

The accuracy of RepeatsDB-lite and therefore the quality of RepeatsDB annotations strongly depends on the quality of the unit library. In particular, similar repeat units in different proteins should be annotated with the same phase, i.e. with the same start/end position of the repeated element and aligned secondary structure. This is especially relevant for studies comparing the position of repeat units with other features or to exploit repeat unit definitions to create profiles (e.g. in Pfam (27)) to detect repeats from sequence in genome-scale analysis. The new version of RepeatsDB focuses on the removal of phase inconsistencies. This is possible thanks to the implementation of a novel protein centric page which allows curators to compare multiple PDB structures mapping to the same protein on a single view and fix errors. The new version of RepeatsDB also introduces a finer classification of repeat regions. Attempts to classify in detail a particular type of TR-containing proteins (28) revealed that RepeatsDB needs at least two additional classification levels. Similarly to the four-level classification schemas used in CATH (2) and SCOP (3), RepeatsDB 3.0 provides ‘Class’, ‘Topology’, ‘Fold’ and ‘Clan’ levels. An additional ‘Family’ level, not yet available, defines groups of homologous repeats within a clan and is defined in collaboration with the Pfam database (27). Finally, in addition to a revised classification, RepeastDB 3.0 includes redesigned web server and interface to improve user experience and data curation. New features allow to compare the position of repeats over different protein structures, to evaluate sequence and structural similarity within a repeated region and navigate the classification.

## PROGRESS AND NEW FEATURES

### Database content

Since its first release, RepeatsDB aimed at the annotation of all these features in repeat proteins, either automatically or through manual curation. The RepeatsDB 3.0 automatic annotation pipeline processes the entire Protein Data Bank with a new version of RepeatsDB-lite (18). The algorithm is based on the repeat unit library, and allows to predict the position of repeat units in the PDB chains, insertions within and between units, as well as the RepeatsDB classification. RepeatsDB supports the visualization of this data by showing the detected repeats in the PDB sequence and structure, allowing navigation of the TR classification and supporting complex queries. The new database version includes several strategies to support the standardisation of repeat phases. (i) We implemented a visualization tool to analyse the structural similarity between units in a region, i.e. the unit similarity matrix. (ii) We compare the unit position with evolutionary sequence features such as Pfam domains (27) and intron/exon structure (29). (iii) We added a unified view of all PDB chains mapped to the same UniProt entry, allowing visualization and comparison of their annotations and visualization of a repeat consensus, i.e. the position of repeat regions in the UniProt entry derived by structural annotation. Finally, we allowed the manual curation of unit positions at the UniProt level, by inspection of the multiple available structures. These reviewed UniProt entries allow the establishment of a common phase and evaluation of TR structural diversity among different PDB structures of the same protein sequence.

### RepeatsDB classification

**Figure 1.**
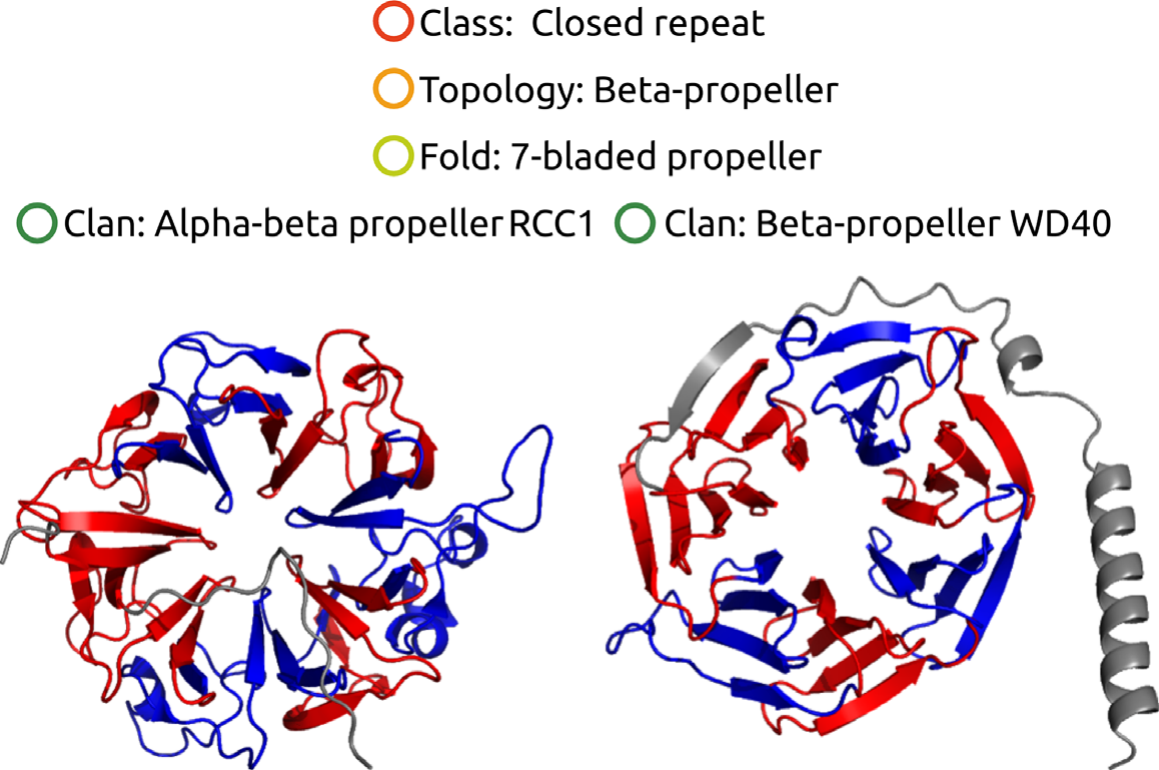
RepeatsDB classification. The new levels of RepeatsDB classification will discriminate finer structural and functional differences. RepeatsDB topology 4.4 includes beta-propeller regions. The folds in topology 4.4 are distinguished by the number of units (in propellers called ‘blades’), while the clans by the specific secondary structure content and the relative orientation of the blades, as well as the overall shape of the region.

Given the increasing number of TR protein structures, we concluded that to classify all structures in a better way, the previous schema with three levels (class, subclass and cluster) had to be extended to five levels (Figure 1). We formulated distinctive characteristics of the additional levels and started to implement these levels in RepeatsDB 3.0. They are: (i) ‘Class’ reflects a general shape, mode of interaction between the repetitive elements and the oligomerization state depending on the repeat length (6). (ii) ‘Topology’ (formerly ‘subclass’) distinguishes a general path of the polypeptide chain and type of the secondary structure in a repetitive unit. (iii) ‘Fold’ is a refinement of ‘topology’, differing in secondary structure arrangement and/or overall structure (e.g. twist) within the repeat. (iv) ‘Clan’, a subfold that groups protein structures having a common sequence motif within the repeat (or part thereof).

An additional fifth level, ‘Family’, will accommodate structures that have a common ancestor based on sequence similarity. Family classification aims at joining the sequence- and structure-based TR classifications of RepeatsDB and Pfam (27) and to support the transfer of evolutionary and functional information through our template-based methods. To address this issue, we extended our collaboration with Pfam (27) in order to improve existing Pfam domains and create accurate models of repeats based on structural information. At the time of writing no clans are annotated at the family level yet as this is work in progress. Pfam information is also included in the annotation of RepeatsDB clans. RepeatsDB clans are generated by structurally clustering units within a fold and comparing the cluster structure with sequence-based information from Pfam. Clusters that are homogeneous both in terms of structural arrangement and Pfam assignment (i.e. each Pfam domain mapping to only one structural cluster) are manually annotated with functional or structural information and included in the classification.

### Data generation pipeline and updates

The starting point for RepeatsDB is the entire PDB (1). At each PDB update, repeat candidates are extracted with RepeatsDB-lite (18) to confirm the presence of repeat regions and provide detailed unit information. PDB chains annotated as containing a repeat region are then clustered at 100% sequence identity. The clusters that map to regions that were already annotated as repeats in previous database releases are automatically added to the database. This pipeline can be automated and will allow regular update of RepeatsDB as well as interoperability with other biological databases.

Clusters mapping to new candidate repeat regions require manual inspection to confirm the presence of repeats in at least one representative group entry. Once this is confirmed by an expert evaluation, clusters are included in RepeatsDB. If the exact position of repeat units is also revised and/or manually annotated, the entry is labeled as ‘reviewed’. The PDB chains detected as containing repeats are then annotated with additional information retrieved from SIFTS (30), to map PDB chain identifiers to UniProt (31) and other biological databases, such as Pfam (27). These data support a comprehensive validation carried out by visual inspection, generating RepeatsDB reviewed entries at the level of the PDB chains or at the level of UniProt entries.

### RepeatsDB website

The RepeatsDB database structure was redesigned to support automatic updates and interoperability with the PDB and UniProt public APIs. Data is however stored locally to prevent broken dependencies and as a MongoDB database. As RepeatsDB data is expected to serve experimentalists as well as bioinformaticians, the website was designed as a multi-tier architecture. It is accessible through a web interface or programmatically exploiting a RESTful architecture. The user interface has been completely redesigned to improve user experience and satisfy both general use and detailed analyses. It retrieves data from public APIs without further processing, allowing accessibility to the same type of data as the web interface to the users of the web server. The web interface is implemented using the Angular and Bootstrap frameworks. Dynamic and interactive elements are developed using D3 (32) for tree visualization, Chart.js (chartjs.org) for histograms visualization, LiteMol (33) for PDB structure visualization, Feature-Viewer (34) to visualize protein features mapped over the sequence, and a custom library as sequence viewer. The interface home page provides direct access to all entries (to the ‘Entry page’ of either PDB chain or UniProt entries) by structural class. For a finer search, the user can visit either the ‘Browse’ page providing access below the ‘class’ level or use the ‘Search’ page for generating complex queries.

#### Browsing and searching data

**Figure 2.**
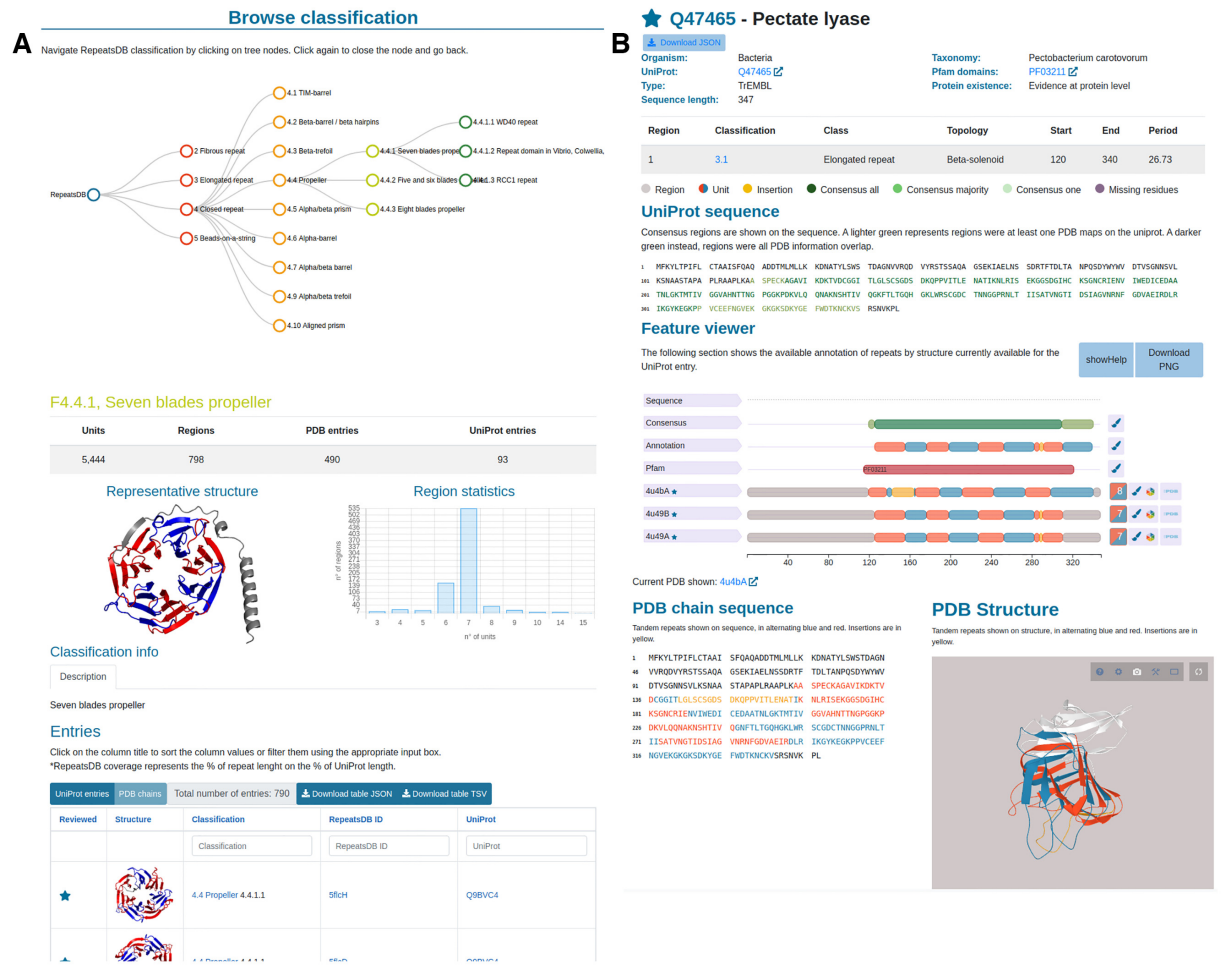
(**A**) RepeatsDB Browse page. This features the classification tree (top) and details of the classification level selected in the tree (bottom). It includes a summary table of the level statistics, image of a representative structure, histogram of unit numbers over per region and a table including all entries belonging to the selected level. (**B**) UniProt entry page. This shows details of the entry and the consensus repeat annotation (top), Feature Viewer with repeat data for all PDB chains mapped to the UniProt entry (center), PDB section showing repeat data on the sequence and structure of the selected PDB (bottom).

The user interface presents an intuitive summary table providing direct access to all entries by structural class directly from the home page, and a search box on the right top for straightforward searches based on UniProt accessions, PDB or RepeatsDB IDs and free text searches. For a finer search, the user can visit either the ‘Search’ page for generating complex queries or the ‘Browse’ page providing full classification access (see Figure 2). The ‘Search’ page allows the user to perform advanced queries against a range of RepeatsDB-specific and third-party search fields. The input can be simple text or numeric (single value or range) according to the field type and multiple queries can be combined by boolean operators (AND, OR, NOT). The ‘Browse’ page provides the entry point for all levels of the new RepeatsDB classification. It contains a representative image and descriptive statistics such as the number of units, regions, PDB and UniProt entries. An extended description of the class or, when available, a link to the Wikipedia annotation, and a histogram showing the number of units per region within the class, topology, fold or clan are also provided.

#### PDB and UniProt entry pages

This version of RepeatsDB introduces two types of entry pages in order to allow different data visualizations (Figure 2). The PDB chain entry visualization is similar to the entry page from previous versions, including basic information about the PDB entry, the summary of detected repeat regions (annotated with start, end, classification) and the position of repeats over sequence and structure. The PDB entry page includes a tab for each repeat region showing the multiple structural and sequence alignment of units. In addition, the novel ‘structural similarity matrix’ allows the visualization of the pattern of similarity between units within a repeat region. The repeat information of different PDB chains mapping to the same UniProt entry is aggregated in the UniProt entry page, newly introduced in RepeatsDB 3.0. This page features basic information about the UniProt entry, its repeat annotation and classification, as well as an interactive Feature-Viewer, showing the position of the consensus repeat region, Pfam domains and all PDB chains mapped to the entry with the position of their repeat units and insertions. The consensus repeat region is derived from the annotation in the PDBs reported in the Feature-Viewer, and colored in increasing shade according to the number of chains that confirm the positional annotation. Missing residues are also reported in this feature. On the bottom, a representative PDB chain is annotated as described in the PDB chain entry page. Different download buttons in both entry pages allow users to retrieve information in different formats.

### RepeatsDB API

RepeatsDB provides programmatic access to perform a search through a RESTful web service API. A single entry can be retrieved by using PDB or UniProt identifiers, while database searches can be performed by specifying query fields directly as URL parameters in the HTTP request. Free text search is also available, retrieving matches for the most common types of biological identifiers or substrings in the protein name. RepeatsDB annotation is available for download in DB (RepeatsDB files), JSON, FASTA and TSV formats. Aiming to make RepeatsDB data more FAIR, we implemented Bioschemas markup using the JSON-LD format in the main and entry pages.

## CONCLUSIONS AND FUTURE WORK

RepeatsDB was first introduced in 2014 with an updated release in 2017. Our continuous classification and annotation effort aims to provide the community with a central resource for high-quality tandem repeat protein characterization. The database has been used in several studies regarding TRs and to benchmark algorithms for the detection of proteins with repeats. The iterative annotation process that bases RepeatsDB update and the interface for the automatic prediction curation (18) will allow a continuous growth and increase in quality of the extensive TR annotation. The main novelties of the presented RepeatsDB release regard (i) the new data visualization, based on UniProt entries and oriented to standardize the annotation of repeat phases and (ii) the addition of two levels in the RepeatsDB classification schema, i.e. folds and clans, representing TRs with similar overall structural arrangement (twist, curve, etc.) and TRs with a common sequence motif, respectively. This classification effort provides the basis for future work. A fine comparison and description of the relationship between the tandem repeat region sequence (*e.g*. from Pfam) and structure-based classifications will provide the toolbox for transferring annotation of TRs from different sources. In addition, uniform TR structural clusters (in terms of evolutionary origin and repeat phase) will provide an additional classification level, the ‘family’ level, and will be exploited for the creation of sequence profiles for use in detecting repeats from sequence in genome-scale analyses (35). Finally, the curation community provided by the RepeatsDB consortium and the MSCA-RISE project ‘REFRACT’ will expand repeat classification and guarantee data quality and long term maintenance.
